# Oxidative Stress in Transthyretin-Mediated Amyloidosis: An Exploratory Study

**DOI:** 10.3390/antiox13080998

**Published:** 2024-08-18

**Authors:** Marco Fiore, Chiara Cambieri, Laura Libonati, Federica Moret, Edoardo D’Andrea, Maria Grazia Di Certo, Claudio Passananti, Francesca Gabanella, Nicoletta Corbi, Matteo Garibaldi, Cristina Chimenti, Maria Alfarano, Giampiero Ferraguti, Silvia Francati, Maurizio Inghilleri, Marco Ceccanti

**Affiliations:** 1 CNR-Institute of Biochemistry and Cell Biology, Via Ercole Ramarini 32, 00015 Monterotondo, Italy; mariagrazia.dicerto@cnr.it (M.G.D.C.); francesca.gabanella@cnr.it (F.G.); 2 Center for Rare Neuromuscular Diseases, Department of Human Neuroscience, Sapienza University of Rome, Viale dell’Università 30, 00185 Rome, Italy; 3 CNR-Institute of Molecular Biology and Pathology, Department of Molecular Medicine, Sapienza University of Rome, Viale Regina Elena 291, 00161 Rome, Italy; 4 Department of Neuroscience, Mental Health, and Sensory Organs (NESMOS), Sant’Andrea Hospital, Sapienza University, 00189 Rome, Italy; 5 Department of Clinical, Internal, Anesthesiologist and Cardiovascular Sciences, Sapienza University of Rome, 00185 Rome, Italy; 6 Department of Experimental Medicine, Sapienza University of Rome, 00185 Roma, Italy; 7 IRCCS Neuromed, 86077 Pozzilli, Italy

**Keywords:** amyloidosis, transthyretin, oxidative stress, FORT, FORD

## Abstract

Transthyretin-mediated amyloidosis (ATTR) is a systemic disease with protein precipitation in many tissues, mainly the peripheral nerve and heart. Both genetic (ATTRv, “v” for variant) and wild-type (ATTRwt) forms are known. Beyond the steric encumbrance, precipitated transthyretin seems to have a toxic effect. In this study carried out in men, we recruited 15 ATTRv patients, 7 ATTRv asymptomatic carriers, 14 ATTRwt patients and 10 young and 13 old healthy controls to evaluate the oxidative stress using FORD (Free Oxygen Radicals Defense) and FORT (Free Oxygen Radicals Test) analyses. ATTRv patients showed reduced FORD compared to ATTRwt and ATTRv asymptomatic carriers. FORD independently predicted the disease stage, with the early stages characterized by the highest consumption. These findings suggest a role for oxidative stress in the early stages of ATTRv.

## 1. Introduction

Transthyretin-mediated amyloidosis (ATTR) is a disease characterized by the extracellular deposition of amyloid fibrils in different tissues, generating a functional impairment. The fibrillogenic process can involve either a wild-type transthyretin in the elderly (ATTRwt) or a genetically mutated protein (ATTRv, “v” for variant). The highest protein synthesis occurs in the liver, with other organs involved in the protein synthesis, such as the brain choroid plexus and retinal pigment epithelium [1,2].

Although ATTRwt is probably more frequent to be still considered a rare disease, at least in the elderly population [3], ATTRv is still a rare disease [4], even if a higher prevalence is described in some areas [5]. The fibrillogenic process includes a dissociation into transthyretin monomers and deposition in the extracellular spaces of systemic organs. The dissociation results in the misfolding of TTR monomers and subsequent aggregation [6].

ATTR, both as a “variant” and “wild-type”, can be considered a gender-related disorder, with males being more frequently affected than females [3,7,8], probably due to the protective role of female hormones [9].

Many strategies have been proposed for ATTRv treatment [10,11]. Hepatic transplantation was the first proposed treatment intended to substitute the genetic profile of the main TTR synthesizer organ. Subsequently, TTR stabilizer drugs were approved both for polyneuropathy and cardiomyopathy [12,13], and new drugs with this action mechanism are going to be approved soon [14]. In the new millennium, TTR synthesis inhibitors have dramatically changed the disease prognosis, stabilizing the disease progression using the small-interference and antisense oligonucleotide mechanisms [15,16].

The pathogenic mechanism of amyloidosis is not just related to the mere steric encumbrance. Many papers have demonstrated a direct toxic effect of amyloid deposits [17], with the activation of pro-inflammatory genes [18], modulation of mitochondrial function, and activation of oxidative stress in the cardiomyocytes [19]. Oxidant mechanisms can also be involved in amyloid aggregation. Though the exact mechanism for TTR deposition is still unknown, for specific mutations, i.e., the most frequent Val30Met, the punctual substitution of an aminoacidic residual was demonstrated to favor the oxidation of 30-Methionine, thus disrupting the β-structure [20]. Myeloperoxidase was demonstrated in amyloid deposits [21]. An in vitro study showed that human Schwannoma cells treated with aggregated TTR increased their release of H_2_O_2_, decreased catalase activity and reduced glutathione levels, thus reducing and consuming the overall cellular antioxidant capacity [22]. In the same way, iPSC-derived neuronal and cardiac cells display oxidative stress and an increased level of cell death when exposed to mutant TTR [23].

Indeed, rising indications suggest a crucial connection between ATTR and oxidative stress. The misfolded TTR may elicit oxidative stress by producing reactive oxygen species (ROS) [17,24], which potentiate protein misfolding, impair cellular constituents [25], and stimulate further amyloid accumulation [24,26]. Furthermore, the oxidative stress activated by ATTR might damage antioxidant defenses, generating a vicious cycle that potentiates ATTR development and tissue injury. Further knowledge of this relationship could be crucial for developing early therapeutic approaches affecting oxidative stress and amyloid deposition in ATTR. Consequently, using biomolecular methods to quantify the levels of free oxygen radicals and the antioxidant capacity in biological samples may offer a direct degree of oxidative stress.

Given the demographic characteristics in the ATTR population [8], we recruited male patients with ATTRv, asymptomatic carriers of TTR mutation (ATTRv asymptomatic carriers), patients with ATTRwt, and healthy controls to check differences in the serum oxidative stress biomarkers by analyzing, for the first time to the best of our knowledge, (i) the serum Free Oxygen Radicals Defense (FORD) and (ii) Free Oxygen Radicals Test (FORT) to disclose a comprehensive assessment of oxidative balance by measuring both the presence of free OH^−^ and the physiological capability in individuals with ATTR to counteract them.

We predict that oxidative stress could be enhanced in ATTR, with a possible consumption of antioxidant systems.

## 2. Materials and Methods

### 2.1. Patients’ Recruitment

In total 15 patients with ATTRv (all with a neurologic and cardiologic mixed phenotype; 5 with V50M mutation, 4 I88L, 4 V142I, 2 F84L), 7 ATTRv asymptomatic carriers (4 with the V50M mutation, 2 V142I, 1 P84L), 14 patients with ATTRwt, 10 young and 13 aged healthy controls were recruited in this observational study. It should be noted that the healthy individuals (controls) are regular blood donors who see donation as a mission and their blood analyses are always checked for eventual alterations that could alter oxidative stress. ATTRwt cardiomyopathy was defined by a Perugini grade 2 or more at the bone scintigraphy, once excluding monoclonal gammopathy [27].

To exclude gender variability, only male subjects were included in all the groups. Patients with ATTRv were classified according to the familiar amyloid polyneuropathy scale in three stages from FAP1 to 3, with asymptomatic carriers considered FAP 0; regarding the cardiologic impairment, an NYHA class was assigned to patients with ATTRv or ATTRwt, with ATTRv asymptomatic carriers and controls considered as NYHA class 1. Every patient with ATTRv underwent a Neuropathy Impairment Score (NIS) evaluation to characterize the neuropathic impairment and a Norfolk questionnaire for the quality of life. The FAP stage and NIS are independent scores, both related to neurological status. In particular, FAP is a functional stage which classifies patients according to their gait impairment. The NIS is a clinical score resulting from the summation of parameters associated with strength, sensibility and reflexes.

The interventricular septum (IVS) thickness was assessed in patients with ATTRv or wt and ATTRv asymptomatic carriers. No patients took anti-inflammatory/antioxidant drugs in the two weeks before the blood sample collection. All of the patients with FAP1 ATTRv or ATTRwt were naïve for RNA-interference therapy [28] or TTR stabilizers [13,29] at the blood collection; patients with ATTRv with FAP stage 3 (N = 3) had experienced disease progression on RNA-interference therapy and discontinued its use at least a year prior to sample collection (RNA-interference therapy is indicated only for FAP 1 and FAP 2 patients).

### 2.2. FORD (Free Oxygen Radicals Defense) and FORT (Free Oxygen Radicals Test) Analyses

We measured the serum oxidative stress status using kits (Callegari, Parma, Italy) to analyze ROS (FORT) and the endogenous defense against free O_2_ radicals (FORD) [30,31]. It should be noted that according to the manufacturer, these tests do not interact also because different serum amounts from the original serum sample were used for the determination. Briefly, the FORT is a colorimetric assay based on the ability of transition metals such as iron to catalyze, in the presence of hydroperoxides (ROOH), the formation of free radicals by the Fenton reaction (reaction 1–2), which are then trapped by an amine derivative, CrNH_2_. The amine reacts with free radicals, forming a colored, long-lived radical cation detectable at 505 nm (reaction 3). The intensity of the color correlates directly to the number of radical compounds and the hydroperoxides concentration and consequently to the oxidative status of the sample according to the Lambert–Beer law [30].

1. R-OOH + Fe^2+^ → RO^•^ + OH^−^ + Fe^3+^

2. R-OOH + Fe^3+^ → ROO^•^ + H^+^ + Fe^2+^

3. RO^•^ + ROO^•^ + 2CrNH_2_ → ROO^−^ + RO^−^ + [CrNH^2+•]^_purple_

According to the manufacturer’s instructions, FORT values below 300 units (U) indicate an optimal condition of oxidative stress, values between 300 and 330 U indicate latent oxidative stress and values superior to 330 U indicate oxidative stress in progress (310 FORT units correspond to 2.36 mmol/L H_2_O_2_–0.26 mg/L H_2_O_2_ eq).

As for FORD, this test uses preformed stable and colored radicals and determines the absorbance decrease proportional to the blood antioxidant concentration of the sample according to Lambert–Beer’s law [31]. In the presence of an acidic buffer (pH = 5.2) and a suitable oxidant (FeCl_3_), the chromogen (which contains 4-Amino-N, N-diethylaniline sulfate) forms a stable and colored radical cation photometrically detectable at 505 nm. Antioxidant compounds in the sample reduce the chromogen’s radical cation, quenching the color and producing a decoloration of the solution proportional to their concentration. The absorbance values obtained for the samples are compared with a standard curve obtained using Trolox (6-Hydroxy-2,5,7,8-tetramethylchroman-2-carboxylic acid), a permeable cell derivative of vitamin E commonly employed as an antioxidant.

1. Chromogen _(no color)_ + Fe^3+^ + H^+^ → Chromogen^•+^ _(purple)_

2. Chromogen^•+^_(purple)_ + AOH → Chromogen^+^ _(no colour)_ + AO

### 2.3. Statistical Analysis

Demographic statistics were expressed as mean ± standard error mean (SEM). Scalar variants were compared with the T-Student or Mann–Whitney U-Test/Kruskal–Wallis according to the distribution, as appropriate; Bonferroni correction for multiple comparisons was applied when multiple hypotheses were tested to reduce type I errors. General linear model (GLM) univariate analysis was used to test regression analysis and analysis of variance for one dependent variable by one or more factors and/or variables. IBM SPSS version 27.0 was used for the data analysis; statistical significance was set as *p* < 0.05.

## 3. Results

The demographic data of the five groups are shown in Table 1. Age at the sample collection was not different between patients with ATTRv vs old controls, ATTRwt vs old controls and ATTRv asymptomatic carriers vs young controls. Patients with ATTRv were younger than those with ATTRwt (*p* < 0.01), as expected.

**Figure 1:**
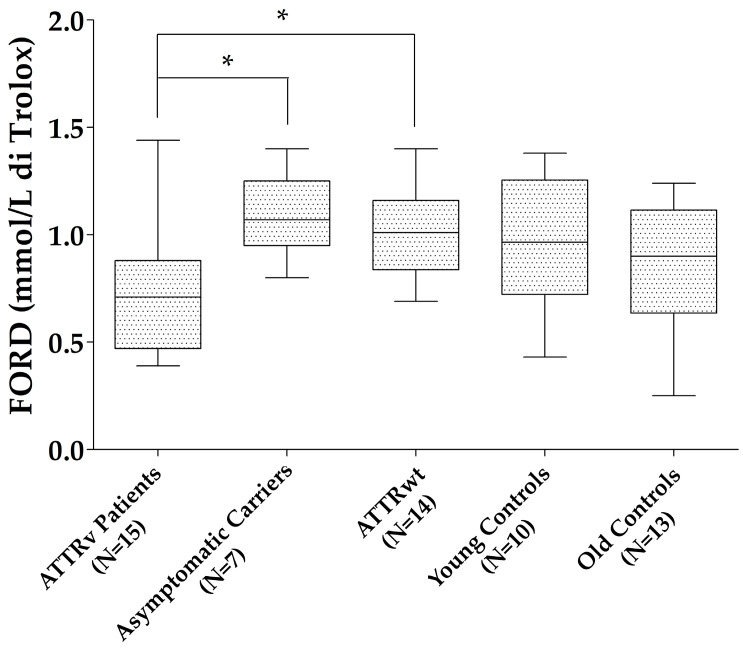
FORD levels in the different groups. * *p* < 0.05. Boxes indicate upper and lower quartiles, and whiskers indicate the minimum to the maximum value.

Patients with ATTRv showed reduced FORD compared to those with ATTRwt (*p* = 0.027) and ATTRv asymptomatic carriers (*p* = 0.023), with a trend to significance compared to old controls (*p* = 0.075). No differences were found for the other oxidative stress markers (FORT units and FORD/FORT) between patients with ATTRv and those with ATTRwt, ATTRv asymptomatic carriers, and old controls, neither between ATTRv asymptomatic carriers and young controls nor between ATTRwt and old controls (Figure 1).

**Figure 2:**
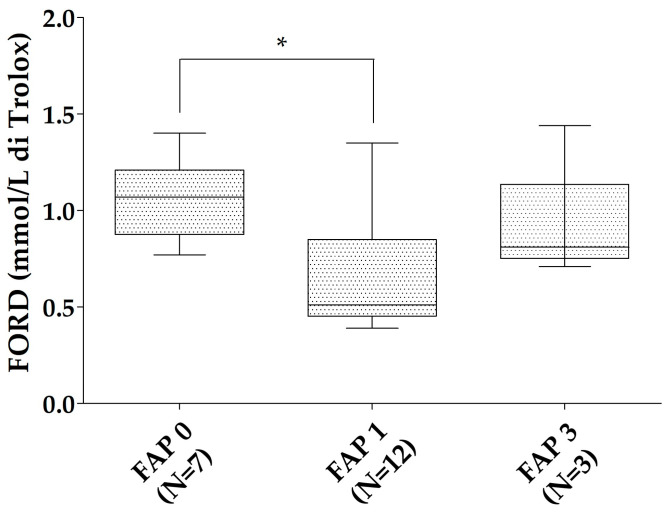
FORD levels in the different FAP stages. * *p* < 0.05. Boxes indicate upper and lower quartiles, and whiskers indicate the minimum to the maximum value.

All of the patients with ATTRv were characterized according to the neurological impairment in the FAP scale. The ATTRv asymptomatic carriers were considered as FAP 0 (N = 7). In the ATTRv patients group, 12 were classified as FAP 1 and 3 as FAP 3. No patient had an FAP 2 stage. FORD was reduced in FAP 1 patients compared to ATTRv asymptomatic carriers (*p* = 0.019). A trend to reduction was found for the FORD/FORT ratio of FAP 1 patients compared to ATTRv asymptomatic carriers (*p* = 0.079—Figure 2). No relationships were found between oxidative stress markers and Norfolk, IVS thickness, and NIS in the patients with ATTRv. No significant differences were found for FORT and FORT levels in different FAP stages when the ATTRv group was divided for single TTR mutations.

One-way ANCOVA was conducted to determine the effect of FORD levels on the FAP stadium, controlling for the NIS scale, FORT, and BMI. The overall model was significant (*p* = 0.003, eta2 = 0.943); FORD was associated with the FAP stage as a significant variable (beta coefficient: −0.541, 95%CI −1.069 to −0.14; adjusted *p* = 0.045) regardless of the NIS, BMI and FORT. Even the NIS was proved to be an independent variable for the FAP stage (beta coefficient: 0.023, 95%CI 0.018 to 0.028; adjusted *p* < 0.01), as expected.

## 4. Discussion

ATTRv is a rare systemic disease, with many organs implicated in the disease progression. The first and most involved organs are the peripheral nervous system, both with the small- and high-diameter fibers [32,33], and the heart. Nevertheless, other organs may be involved, such as the kidneys, eyes, brain and many others [34]. The newly available drugs effectively stabilize the progression of the heart and peripheral nervous system; some concerns remain about the effectiveness of the drugs on other organs [35,36].

In the era of effective drugs, the pre-symptomatic stage of the disease and the physio-pathological mechanisms have seen a surge in terms of interest. Some serum markers, notably light chain neurofilaments (NfLs), have been proposed and are currently used to follow up ATTRv asymptomatic carriers [37,38,39]. Despite their utility in the clinical setting, NfLs represent the final stage of peripheral nervous system damage and are not directly related to the physio-pathological mechanisms of disease onset.

Beyond the steric encumbrance after extra-cellular deposition, the amyloid also induces oxidative stress through gene modulation [19,20,21]; aggregated TTR itself seems to increase the release of H_2_O_2_, thus consuming the antioxidant cell capacity [22]. This could be a very early pathogenic moment, even in vivo. The results of our study demonstrate a FORD reduction in patients with ATTRv compared to ATTRv asymptomatic carriers and those with ATTRwt, with a trend to significance compared to old healthy controls. Interestingly, FORD has reduced in the early FAP 1 stage of patients with ATTRv compared to ATTRv asymptomatic carriers, with a trend to significance even for a FORD/FORT ratio reduction in the same comparison. No significant differences were found for FORD and FORT levels in different FAP stages when the ATTRv group was divided for single TTR mutations, probably because the small sample of the single groups impaired statistical power.

FORD reduction was demonstrated to determine the FAP stage, even independently from other strong variables such as the NIS and BMI, thus confirming its strong role in the early stages of the disease.

Oxidative modifications of lipids and proteins have been reported in colon tissues and protein nitration in the nerves of patients with ATTRv [40,41]. Our data confirm anti-oxidant consumption in patients with ATTR, previously demonstrated in in vitro experiments [22,23]. In these experiments, catalase activity and glutathione were reduced in cells cultured with aggregated TTR, with an increase in H_2_O_2_ production. The authors [41] conclude that pro-oxidative factors can lead to the depletion of catalase activity and glutathione in cells, and such depletion can further result in increased oxidative damage in nearby cells. Other authors demonstrated the cytotoxicity induced by TTR fibrils binding to the receptor for advanced glycation end products (RAGE) in the peripheral sural nerve, with the local activation of pro-inflammatory cytokines such as TNFα and IL-1β and the induction of the transcripts for iNOS. The same TTR fibrils–RAGE binding could induce Caspase-3-dependent apoptosis. This model could effectively explain some of the systemic effects of ATTR and fits well with our data.

There was no relationship between FORD and parameters associated with disease progression (NIS and IVS thickness), confirming a role for antioxidant system consumption only in the early pathogenic stages. Moreover, even if in a very limited sample, FAP 3 patients’ FORD levels were not significantly reduced compared to ATTRv asymptomatic carriers. Finally, patients with ATTRv also showed reduced FORD compared to those with ATTRwt. These last patients typically have a longer disease history than patients with ATTRv, with a slower disease progression.

This exploratory study has some limits: the small sample size and the FAP stage distribution can impair definitive conclusions. Nevertheless, ATTRv is a rare disease, and the multiple comparative groups and gender homogeneity can represent a strength. Indeed, ATTR, both in the wild-type and variant forms, can be considered a gender-associated disease, with males accounting for almost 80% of patients [7,8,42]. Additional studies are needed to validate these data further and determine the possible role of different TTR mutations in oxidative stress and antioxidant consumption.

Another potential limit of the present investigation is the fact that many factors may bias oxidative stress in both patients and healthy controls such as the consumption of drugs, alcohol, anti-inflammatory compounds, and/or a diet rich/poor in vegetables containing polyphenols with antioxidant abilities. However, it should be noted that (i) anti-inflammatory/antioxidant drugs are not usually indicated for ATTR treatment and were not taken by our patients and (ii) healthy individuals (controls) are regular blood donors who see donation as a mission and their blood analyses are always checked for eventual alterations. Furthermore, patients are aware of the problems induced by the disorder, including the associated outcomes due to an unbalanced/unhealthy diet.

As for inflammation and oxidative stress in ATTR, Diflunisal, a non-steroidal anti-inflammatory drug (NSAID), has shown effects in the treatment of ATTR amyloidosis [43,44,45]. Diflunisal exerts its useful effects primarily through its capability to stabilize the TTR tetramer, thus inhibiting the separation of TTR into monomers. Inflammation is a common reaction to amyloid deposition and can potentiate tissue damage by increasing oxidative stress. By decreasing inflammation, Diflunisal may help to counteract some of the secondary injuries caused by the inflammatory response to amyloid deposits. This anti-inflammatory/oxidative stress action could play a role in the beneficial effects observed in patients with ATTR amyloidosis.

## 5. Conclusions

In conclusion, our findings disclosed a noticeable reduction in FORD levels in patients with ATTRv compared to patients with ATTRwt and ATTRv asymptomatic carriers, representing a compromised antioxidant defense mechanism in the variant form of the disorder. Interestingly, FORD levels were discovered to be an independent determinant of the neurological functional stage in patients with ATTRv, with the early phases showing the main consumption of antioxidant defenses. This indicates that oxidative stress plays a serious role in the initial stage of ATTRv pathogenesis, potentially contributing to the development of the disorder.

The detected oxidative stress in patients with ATTRv underscores the need for further examination into therapeutic strategies aimed at bolstering antioxidant immunities. Directed antioxidant therapies could hypothetically delay the disease onset by mitigating the oxidative injury that seems to be prevalent in the early phases of ATTRv. Moreover, the noteworthy difference in oxidative stress biomarkers between patients with ATTRv and those with ATTRwt suggests different pathogenic mechanisms underlying these disorders. The disease onset in ATTRwt is more subtle and oxidative injury is probably less of a determinant.

Moreover, the presence of ATTRv asymptomatic carriers in our study provides important information about the pre-symptomatic stage of ATTRv. Further, oxidative stress may begin to gather even before clinical symptoms, underscoring the potential for early intervention strategies in people identified as carriers through a genetic assessment.

Overall, our study supports the hypothesis that oxidative stress is a crucial player in the pathophysiology of ATTRv, particularly in its early phases. The significant reduction in FORD levels among patients with ATTRv suggests a sensitive susceptibility to oxidative damage, which could aggravate the toxic actions of precipitated transthyretin. A future investigation should aim to clarify the oxidative stress molecular mechanisms and study hypothetical antioxidant treatments that could delay, counteract, or prevent the evolution of ATTRv. By understanding the function of oxidative stress in ATTR, we may disclose more effective and directed therapeutic interventions, finally improving outcomes for individuals suffering from this debilitating disorder.

## Figures and Tables

**Table 1:** Demographic data and oxidative stress-related parameters. M: median. Q1–Q3: first and third quartile. NIS: Neuropathy Impairment Score. QoL: quality of life. BMI: body mass index.

	Patients with ATTRv	ATTRv Asymptomatic Carriers	ATTRwt	Young Controls	Old Controls
Age, m (Q1–Q3)	69 (62−76)	45 (38−59)	84 (80.7−86)	55.5 (41.5−59.7)	75 (70−81.5)
NIS, m (Q1–Q3)	26 (13.2−33.2)				
Norfolk QoL, m (Q1–Q3)	48 (23.7−74.7)				
BMI, m (Q1–Q3)	24.2 (22.2−24.6)	25 (23.9−26.6)	24.2 (23.2−25)		
FORT Units, m (Q1–Q3)	1.47 (1.2−2)	1.9 (1.2−2.7)	1.8 (1.2−2.3)	1.7 (1.2−2.2)	1.2 (1.2−2)
FORD (mmo/L), m (Q1–Q3)	0.7 (0.47−0.88)	1.07 (0.95−1.25)	1.01 (0.84−1.16)	0.96 (0.72−1.25)	0.9 (0.63−1.11)
FORD/FORT, m (Q1–Q3)	0.38 (0.29−0.66)	0.56 (0.37−0.95)	0.53 (0.39−0.8)	0.52 (0.43−0.76)	0.56 (0.37−0.68)

## Data Availability

All the data will be available upon reasonable request to the corresponding author.
